# Community-based mental health and well-being interventions for older adults in low- and middle-income countries: a systematic review and meta-analysis

**DOI:** 10.1186/s12877-022-03453-1

**Published:** 2022-09-29

**Authors:** Clarissa Giebel, Nipun Shrestha, Siobhan Reilly, Ross G. White, Maria Isabel Zuluaga, Gabriel Saldarriaga, Ginger Liu, Dawn Allen, Mark Gabbay

**Affiliations:** 1 Department of Primary Care & Mental Health, University of Liverpool, Liverpool, UK; 2 NIHR ARC NWC, Liverpool, UK; 3 NHMRC Clinical Trials Center, University of Sydney, Sydney, Australia; 4 Centre for Applied Dementia Studies, Bradford University, Bradford, UK; 5 School of Psychology, Queen’s University Belfast, Belfast, UK; 6 National Faculty of Public Health, Universidad de Antioquia, Medellín, Colombia

**Keywords:** Ageing, LMIC, Mental health, Interventions, Therapy

## Abstract

**Background:**

Mental health support is often scarce in low- and middle-income countries (LMIC), with mental health stigmatised. Older adults are some of the most vulnerable members of society and may require specific types of mental health support. The aim of this mixed-methods systematic review and meta-analysis was to explore the types, components, and efficacy of psychosocial community-based mental health interventions for older adults (aged 60 + years) residing in LMIC.

**Methods:**

Six databases were searched in August 2021. Studies published since 2000 were included if they collected primary quantitative or qualitative data on community-based interventions for improving mental health for older adults residing in LMICs, focusing on improving mental health and well-being outcomes. Full texts were screened by two researchers.

**Results:**

From 24,378 citations identified, 40 studies met eligibility criteria. Across 12 countries, interventions were categorised into those focusing on (1) Established forms of psychological therapy; (2) Exercise; (3) Education; (4) Social engagement; (5) Multi-component. Most interventions were effective in reducing levels of depression, anxiety, and improving well-being, including reminiscence therapy, different types of socialising, and breathing and laughter exercises. Some interventions reported no benefits and those that did at times failed to report continued benefits at follow-up. Given the variations in intervention type and delivery, cultures, and outcome measures used, underpinning factors of intervention success or failure were difficult to establish.

**Conclusions:**

Psychosocial interventions for older adults in LMIC need to be adapted to local contexts depending on culture and population needs. Existing interventions and their components can be used as a foundation to produce adapted and multi-component interventions, to tackle growing and inadequate mental health care provision in LMIC.

**Trial registration:**

The review protocol was registered on PROSPERO [CRD42021271404].

**Supplementary Information:**

The online version contains supplementary material available at 10.1186/s12877-022-03453-1.

## Background

Appropriate mental health care is often limited in low- and middle-income countries (LMIC) [1]. This is mostly the result of limited availability of mental health care services, or associated stigma in different cultures, with families preferring to care for their relative themselves as opposed to openly sharing a mental health problem in their community [2, 3]. Further barriers include resource and administrative barriers, information and knowledge barriers, and policy barriers [4].

Older adults living in LMICs can be particularly affected by poor mental health. Older adults in these countries are often amongst the poorest members of society, without pensions or alternative consistent income, thus relying on younger family members for basic necessities and housing [5, 6]. Research indicates the need for more diverse approaches to support older adults beyond heavy reliance on family as a support system, which can create vulnerabilities for older adults [5]. This is particularly important given that older adults are the fastest growing population group globally and thus adequate support systems need to be in place [6].

This can leave many mental health issues unaddressed, and highlights the need for providing different types of mental health support from those provided solely by health care providers. Stigma is often a significant barrier to accessing care for mental health needs [2]. One way of addressing this barrier can be by providing support outside of dedicated health services which is integrated into the local community and local contexts. There is a growing evidence base on such psychosocial, community-based interventions in various different countries, delivering support to older adults via established forms of intervention such as Cognitive Behavioural Therapy [7, 8] or educational interventions [9, 10].

Whilst there is a clear need to address the mental health of older adults in LMICs, and a growing evidence base exploring the effects of different psychosocial interventions, there is no systematic review of the evidence to date. Instead, systematic reviews have focused on children or working-age adults with specific conditions [11, 12], or digital interventions for mental health in the adult population [13]. An umbrella review by Barbui et al. (2020) [14] reported on the effects of psychosocial interventions on various mental health outcomes in adults living in LMICs, providing overall support for the efficacy of non-pharmacological interventions. However, neither their review, nor the others we identified have specifically focused on older adults – a particularly vulnerable population group which has been affected to a great extent by the COVID-19 pandemic both in terms of their physical and mental health [15].

The aim of this mixed-method systematic review and meta-analysis was to explore the types, components, and efficacy of psychosocial community-based mental health interventions for older adults residing in LMIC. The meta-analysis specifically aimed to look at the most common type of mental health outcome, which is depression. With no existing review conducted on this topic to date, findings from this review can help identify best practices of effective global mental health interventions and their components, to develop and adapt them for older adults in different settings and contexts.

## Methods

This mixed-method systematic review and meta-analysis was conducted adhering to the guidelines outlined by the PRISMA checklist. A prospective protocol was registered on PROSPERO [CRD42021271404]. Two unpaid carers were involved in interpreting the findings.

### Study selection

Studies were eligible for inclusion if the intervention being evaluated was designed to improve the mental health and/or wellbeing of older adults aged 60 + years living in a low- and middle-income country as defined by World Bank at the time the review was conducted. The mental health promotion intervention considered in this review included but not limited to studies addressing levels and diagnoses of depression, anxiety, loneliness, social isolation, quality of life, social functioning, and mental well-being. For the purposes of this review, mental health and well-being interventions were defined as any psychosocial intervention that aims to improve the mental health or modify factors that are responsible for poor mental health. The intervention should focus on the prevention, promotion, and treatment of mental health and well-being. These included non-pharmacological, psychotherapy, and psychosocial interventions, but excluded pharmacological interventions. The quantitative outcomes considered in this review were indicators of negative mental health such as depression, anxiety, psychological distress, suicidal behaviour and positive mental health such as resilience, emotional wellbeing, and quality of life. Similarly, the qualitative outcomes considered in this review were facilitators, barriers and factors affecting the effectiveness of mental health interventions for older adults residing in LMICs.

For assessing the effectiveness of interventions and their core components, we included randomized controlled trials, cluster randomized controlled trials, and quasi-experimental study designs. For assessing the facilitators and barriers of interventions, we included qualitative study designs such as ethnography, phenomenological approaches, grounded theory studies and qualitative process evaluations.

Studies were excluded if they were: not published in a peer-reviewed journal; did not include data on older people aged 60 + years separately from others; published before 2000; published in languages other than English and Spanish; focused on physiological/pharmacological interventions or complementary/alternative medicinal procedures (e.g., acupressure or acupuncture); were not conducted in the community; were not conducted in an LMIC.

### Searches and search strategy

We searched the following databases from January 2000 to the 5^th^ of August 2021: Pubmed, CINAHL, Embase, PsycINFO, Scopus, and Web of Science. The detailed search strategy can be found in Additional file 1: Appendix 1.

### Screening and selection

Titles and abstracts retrieved from the database searches were imported to EndNote and duplicate records were removed. At Stage 1, a study author (NS) assessed the remaining records against inclusion criteria by checking the title and abstract. A 10% random sample of papers were checked for appropriate inclusion/exclusion by another author (CG). At Stage 2, full text articles were sought for studies that were deemed suitable at Stage 1 and assessed against the inclusion criteria by NS and CG independently. Any discrepancy in the judgement between the authors were sorted by discussion. Reference list of included studies and similar review articles identified during screening were also screened to identify further studies for inclusion.

### Quality assessments

The quality of included studies was rated by a study author (NS) for quantitative studies, and by a different study author for qualitative studies (CG). The Quality Assessment Tool for Quantitative Studies [16] was used to assess the quality of quantitative studies. The studies were rated overall strong when the study had no weak ratings for any domains, including study design, confounders, blinding, data collection methods, withdrawal and dropouts, intervention integrity and analysis appropriate to the question. Similarly, the studies were rated overall moderate with only one weak rating for the eight domains and weak rating when study had two or more weak ratings for two or more domains, or the study had a weak rating for the study design domain.

To assess the qualitative studies the Critical Appraisals Skills Programme (CASP) [17] tool was used. Quality assessments did not influence study selection but was used in guiding the discussion of findings and drawing conclusions.

### Data extraction

Data were extracted using excel sheets designed to extract data from quantitative and qualitative studies separately. The data extracted from quantitative studies included study design, country undertaken, aims, studied outcomes, sample size, participant characteristics, and intervention characteristics. Means, standard deviations and sample sizes were extracted for the intervention and control groups. Similarly, for qualitative studies study objective, guiding framework, data collection and analysis methods and key themes were extracted.

### Synthesis

This systematic review pooled the effect of different categories of psychosocial intervention on depression in older adults in a pairwise meta-analysis, with adequate data for other outcomes not available. One researcher extracted the data, which was discussed with all research team members. Studies were pooled under the following categories: Psychological therapy interventions (subcategories: Cognitive behaviour therapy, Reminiscence therapy and Other forms of therapy), Exercise interventions, Educational interventions, Social engagement interventions, and Multi-component interventions. Only randomised control trials (RCT), cluster randomised control trials (cRCT), and quasi experimental controlled designs were considered for estimating a pooled effect estimate. Post-intervention means and standard deviation for the longest follow up were used in calculating the pooled effect size using the generic inverse-variance method [18]. The studies were heterogenous in terms of intervention components and outcomes measurement, and therefore random‐effects model was used to calculate pooled effect sizes [19]. The pooled effect size in the meta-analyses was reported as standardised mean difference (SMD), as different instruments were used to assess depressive symptoms in the included studies. Three cluster‐RCTs in the meta-analysis that did not account for clustering in their results were adjusted, assuming a large intra‐cluster correlation coefficient of 0.10 [20]. For two studies that included two arms of the same study [21, 22] in the same meta-analysis, the number of participants in the control group was halved to avoid double counting [23]. The extent of heterogeneity was assessed using the *I*^*2*^ statistic with observed value 0%–40% judged likely not important; 30%–60% moderate heterogeneity; 50%–90% substantial heterogeneity; and 75%–100% judged considerable heterogeneity [24]. All statistical analysis were conducted using STATA (StataCorp LLC, USA).

Findings from qualitative studies were narratively synthesised for each respective intervention type.

### Public involvement

Two unpaid carers from the UK were involved in interpreting the findings and in the dissemination. One was a former carer for her mother with dementia, and one was a current carer for her elderly mother with mental health issues. Both unpaid carers attended team meetings and read through the manuscript drafts. Both carers agreed with the interpretation of the findings, and thus ensured their real-life relevance to those with lived experiences.

## Results

### Overview of included studies

**Fig. 1:**
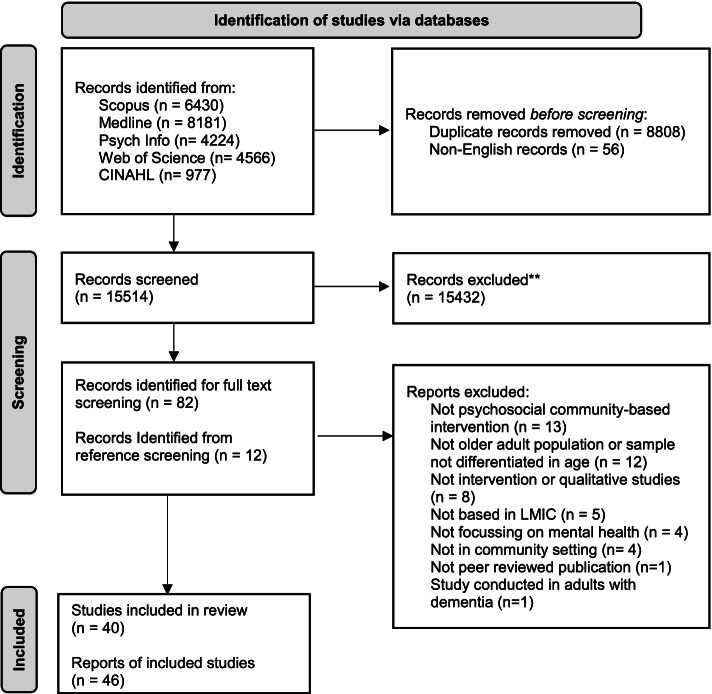
PRISMA Flowchart diagram of study selection

The searches yielded 24,378 citations, with 15,514 hits after duplicates and non-English records were removed. After Stage 1 screening, 95 articles were identified for full text screening. After Stage 2 screening, 40 studies from 46 papers met the inclusion criteria (see Table 1 and Fig. 1) and were included in this review. The studies were conducted in Bangladesh (*n* = 1), Brazil (*n* = 2), China (*n* = 9), Dominican Republic (*n* = 1), India (*n* = 4), Indonesia (*n* = 3), Iran (*n* = 6), Malaysia (*n* = 2), Mexico (*n* = 1), Philippines (*n* = 2), Thailand (*n* = 5) and Turkey (*n* = 4). The interrater agreement for Stage 1 and Stage 2 are 99.3% and 83.7%, respectively. Out of the 40 included studies, 16 were randomized controlled studies [8, 21, 22, 25–37], three cluster randomised control studies [38–40], 12 quasi experimental controlled studies [9, 10, 41–50] and five pre-test post-test studies [7, 51–54]. Three studies [55–57] were RCTs with an embedded qualitative study for the evaluation of intervention implementation. However, for one study, only the result of the qualitative part was available [56]. The final included study was a quasi-experimental controlled study with an embedded qualitative study for the evaluation of intervention implementation [58].

**Table 1:** Characteristics of included studies

Author/year	Intervention description	Country	Type of study/Study design	Population group (sample size, age ranges)	Length and frequency of intervention	Comparison group	Outcomes and assessment methods	Follow up time point	Findings
**Therapy**									
*** Established forms of short-term therapy***									
Siviş and Demir (2007) [58]	Reminiscence therapy program	Turkey	quasi-experimental, controlled study and focus group interview with the intervention group participants Focus group interviews	10 participants with 5 in each group. Mean age: intervention 68 years and control 73 years	Not reported	No intervention	LSI A – Life satisfaction Analysis approach for qualitative data not reported	post intervention	No significant difference between post-test Life Satisfaction scores of older adults in intervention group compared to control group Participants reported positive feedback regarding the group experience, e.g., group’s role in facilitating interaction and friendship among participants, enhancing a more positive self-image and leading to enjoyment and pleasant feelings
Cherian (2019) [7]	Reminiscence therapy	India	pretest–posttest study	50 older adults aged 60–80 years residing in greater Noida, Uttar Pradesh	Not reported		GDS long form—Depression	not reported	significant reduction in depressive symptoms
Efendi et al. (2020) [8]	Cognitive behaviour therapy (CBT)	Indonesia	Randomised control trial	90 older adults > 60 years old living in the post-earthquake Lombok area experiencing PTSD based on the Clinician-Administered PTSD Scale (CAPS-5), GDS ≥ 5 and MMSE ≥ 24. 45 respondents in the intervention group and 45 respondents in the treatment group	Not reported	no intervention	Clinician-Administered PTSD Scale (CAPS-5)—post-traumatic stress disorder; GDS – depression and WHO Quality of Life-BREF – Quality of Life	6 weeks	decrease in post-traumatic stress disorder and depression scores, significant improvement in quality-of-life components
Saisanan Na Ayudhaya et al. (2020) [46]	Behavioural activation sessions delivered subsequently included (1) activity monitoring- examining the effect of specific activities on mood, (2) activity scheduling-developing a plan to increase pleasant activities, and (3) modification-utilizing problem-solving to alter contextual problems that may be eliciting or maintaining depressed mood	Thailand	quasi-experimental, controlled study	82 older adults aged 60 years and above from two subdistricts of Muang district of Samut Songkram Province who were diagnosed with subthreshold depression GDS score between 13 and 24. 41 older adults from each district was enrolled into the study	12 weeks	Regular physical examinations, review of current health symptoms, and psychoeducation delivered by the local mental health nurse	Thai GDS- Depression; DASS- Anxiety	3, 6,and 9 months follow up	significant reductions in the TGDS score and DASS compared to usual care-only group. Lower TGDS and DASS depression and stress scores maintained up to 6 months. Reduction in DASS anxiety score maintained only for 3-month post-intervention
Li et al. (2021) [30]	group reminiscence therapy based on Chinese traditional festival activities. participants attended a four-hour intervention session	China	Randomised control trial	64 Chinese rural older adults (aged 65.70 ± 3.69 years) living alone with 32 individuals each in the intervention group and the wait-list control group	one session each month for 8 months	no intervention	Perceived stress scale—Perceived stress; UCLA loneliness scale—loneliness	3 months	significantly decreased the perceived stress and loneliness of rural older adults living alone in intervention group compared to control group post intervention and at 3 months follow up
Yujia et al. (2021) [36]	group reminiscence therapy intervention in combination with physical exercise	China	Randomised control trial	130 older adults aged 60 years and above from communities in Xiangtan City and Changsha City of Hunan Province, with 65 people in each group	8 weeks	listened to 4 routine health lectures	Spirituality Index of Well-Being—spiritual well-being; ULS Loneliness Scale—loneliness and Brief Resilience Scale – resilience	post intervention (8th week)	Reduction in loneliness and improvement in the spiritual well-being and resilience in the intervention compared to control group
Sutinah (2020) [47]	Psychoeducation therapy was done first and then followed by reminiscence therapy in the next day	Indonesia	quasi-experimental, controlled study	72 older adults in the Simpang Kawat Village, Jambi Indonesia. 36 in the intervention group and 36 in the comparison group. The average age of participants in the intervention and control groups was 68 years	Psychoeducation therapy:5 sessions with 5 meetings, and each session for 45–60 min Reminiscence therapy: 5 sessions with 9 meetings and each session for 75 min. The intervention lasted for 6 weeks	reminiscence therapy	Indonesian version of GDS—depression	post intervention	Reminiscence therapy alone or in combination with psychoeducation therapy effective in reducing depression. The combination of reminiscence and psychoeducation therapy was much more effective than reminiscence therapy alone
Yuan et al. (2020) [57, 59]	Adapted Cognitive behaviour therapy delivered by trained lay health workers	China	Randomised control trial Focus group and in-depth interviews	50 older adults (age: mean 70.5 ± 5.6 years) with Geriatric depression scale score > 9: 24 to the Cognitive behaviour therapy group and 26 to the Control group	Eight sessions	usual care	GDS– depression; Self-rating Anxiety Scale – anxiety and WHO Quality of Life-BREF—social relationships Qualitative data analysed based on thematic framework was developed and agreed on by consensus	week 4 and week 8 (or after the eighth session)	Cognitive behaviour therapy reduced more Geriatric Depression scores over 8-week follow-up compared with usual care The village doctors stressed the importance of role-playing and using instructive manuals in the training. Proper supervision was also a key component of the program. Cultural and political factors facilitated the elders’ access to mental health services. Challenges included a lack of real therapy (in contrast to role-playing) demonstrated in the training and lack of a step-by-step manual based on different types of problems encountered
Viguer et al. (2017) [48]	reminiscence program conducted by a trained psychologist	Dominican Republic	quasi-experimental, controlled study	168 healthy older adults aged 60 years and above recruited through four healthcare centers with no clinical depression (determined by a score of 14 or less on the Geriatric depression scale Spanish version). 84 in each group	10 weekly group sessions lasting two hours each	No intervention	Spanish version of GDS– depression; LSI-A – life satisfaction; Spanish version of the Psychological Well-Being Scales – psychological well-being	post intervention and at three months follow up	significant increases in the time-group interaction, life satisfaction, and psychological well-being measures, and decreases in depressed mood, after treatment. The effects remained after three months in the case of life satisfaction and some dimensions of psychological well-being, but they were lower on depressed mood
Xie et al. (2019) [34]	Modified behavioural activation treatment and regular treatment	China	randomised control trial	Eighty rural left-behind older adults, aged 60 years and above in Yankoutown town of Lengshuijiang City, Hunan Province who had a GDS score between 11 and 25. 40 participants in each group	8 weeks	received regular treatment	GDS—long form – depression; Beck Anxiety Inventory—anxiety and OHQ- happiness	post-intervention, and at 3 months post-intervention	GDS and BAI scores decreased significantly, but the scores of OHQ increased significantly in the intervention group. The reduction in depression symptoms after the intervention was maintained at the 3-month follow-up
Zhou et al. (2012) [40]	health education and group reminiscence therapy by trained community nurses	China	cluster randomised control trial	129 older adults (8 communities) with 62 participants (4 communities) in the intervention group (4 communities) and 67 participants in the control group. average age of participants was 69.4 years	Once a week, for 90–120 min per session, and lasted for 6 weeks	three health education sessions—one every 2 weeks lasting 30–45 min each	GDS -depression; Self-Esteem Scale – self-esteem and Affect Balance Scale – affect balance	postintervention (6 weeks)	Depression scores in the intervention group decreased significantly compared to those in the control group. Scores on the positive affect subscale and affect balance in the intervention group increased significantly higher than control group, and scores on the negative affect subscale decreased significantly lower than control group
*** Other forms of therapy***									
Amigo and Mariati (2020) [51]	Classical music versus music-video therapies	Indonesia	pretest–posttest study with two intervention groups	24 older adults aged 60 years and over who experienced stress (Depression Anxiety Stress Scale score > 14), with 12 older adults in each group			DASS—stress	not reported	Significant effectiveness of both music and music video therapy in reducing stress in older adults
Goksin and Asiret (2021) [29]	Progressive muscle relaxation that involves the controlled contraction and relaxation of large muscle groups in the human body along with regular breathing	Turkey	Randomised control trial	49 elderly women aged 65 and over who were not diagnosed with dementia or psychiatric illness (21 intervention and 28 controls) from a family health centre	28 min sessions three times a week for 8 weeks	no intervention	GDS– depression	post intervention (8th week)	a significant difference in the mean depression scores in the intervention group
Dias, Azariah, Anderson, et al. (2019) [55, 60–63]	Lay counsellors provided problem-solving therapy, brief behavioural treatment for insomnia, education in self-care of common medical disorders such as diabetes, and assistance in accessing medical and social programs	India	Randomised control trial semi structured, in-depth interviews with participants in the intervention arm	181 older adults (≥ 60 years) with subsyndromal depressive symptoms at rural and urban primary care clinics in Goa. with 91 participants in the intervention group and 90 in control group	Six intervention sessions, 30 to 40 min in length that spanned 6 to 10 weeks including 2 booster sessions, 1 each at months 7 and 10	Care as usual	Mini International Neuropsychiatric Interview 6.0; Depression (GHQ-12) Framework analysis (qualitative data)	12 months	Incident episodes of major depression lower in the intervention. The incidence of depressive symptoms was also less Participant Perceptions of the Psychoeducation and Active Coping Strategies, Engagement with the Lay Counsellor, Coping with Physical Health Issues, Engaging in More Pleasurable Activities, Improving Sleep Quality, Using Strategies to Reduce “Tension”, Where the Intervention Was Not Perceived to Be Helpful and Participant Recommendations
Esmaeilzadeh and Oz (2020) [52]	Psychosocial care intervention delivered once a week group meeting session in total of nine sessions. The intervention used visual methods, question answer and discussion technique, homework, and warming games to address emotional, social, and physical problems that the elderly faced	Turkey	pretest–posttest study	44 older adults who are 65-year and above registered in the elderly day care center	each session lasted approx.. 2 h		GDS short form – depression; Turkish version of the WHO Quality of Life Instrument—Health-Related Quality of Life; UCLA loneliness scale—loneliness	post intervention	significant reduction in loneliness and improvement in quality of life but no significant reduction in depression
**Exercise**									
Ansai and Rebelatto (2015) [21]	Multicomponent training session: warm-up using cycle ergometer; aerobic exercise using cycle ergometer; strength exercises of major muscle groups; balance activities; and cool-down exercise. Resistance training group carried out three sets of 10–12 maximal repetitions, with moderate speed and 1-min resting periods between sets	Brazil	Three arm randomised control trial	69 community-dwelling older adults aged 80 years and older from São Carlos with 23 in each group	16 weeks and included three 1-h sessions per week on non-consecutive days	No intervention	GDS—Depression	Post intervention (16 weeks)	No significant differences between groups on Geriatric depression scale
Azizan and Justine (2016) [41]	Exercise behaviour group: group-based exercise followed by behavioural program Exercise group: only the exercise training. conducted	Malaysia	quasi-experimental, controlled study	63 older adults aged 60 and over recruited from three different villages. (a) exercise and behavioural program group (*n* = 18), (b) exercise only group (*n* = 23), and (c) control group (*n* = 22)	Exercise: three sessions per week, each session of 1 h for 6 weeks Behavioural programme: two times per week for 5 weeks	advised to continue with their normal routines	Malay version of the GDS – Depression; SF-12 Health Survey – Health-related quality of life	12 weeks and 24 weeks	Significant main effect only in the level of depression among the three groups for Health-related quality of life, a significant main effect was found on the physical and mental component score
Borbon-Castro et al. (2019) [42]	In a multidimensional exercise program, exercise classes were offered 5 days a week for 12 weeks with a total of 60 sessions. Each session lasted for 60 min, including a warm-up, a variety of exercises, and cool-down. The 12-weeks exercise sessions were divided in to six modules which increased in intensity every two weeks	Mexico	quasi-experimental, controlled study	45 older adults living in the urban community, intervention group (*n* = 23) and control group (*n* = 22). The mean age was 67.7 years for intervention and 66.6 for control group	12 weeks	advised to continue performing the activities of the center in which they were registered	GDS—Depression	1 week after the conclusion of intervention	Depression decreased in the intervention group
Ibrahim et al. (2021) [53]	daily virtual group exercise	Malaysia	pretest–posttest study	Elderly aged 60 years and above recruited from the Promoting Independence in Seniors with Arthritis pilot cohort	4 weeks		Hospital Anxiety and Depression Scale – Anxiety and depression	post intervention	No significant difference in anxiety and depression scores before and after intervention
Moraes et al. (2020) [22]	Aerobic training group performed aerobic exercise on stationary bikes or treadmills. The strength training group performed exercises for the major muscle groups	Brazil	Three arm randomised control trial	27 outpatients from the Center for Alzheimer’s Disease and Related Disorders from the Institute of Psychiatry at the Federal University of Rio de Janeiro. 9 in each group	Both groups had to perform 30 min of moderate intensity physical exercise and had to attend at least 75% of the 24 sessions in 12 weeks	30 min of low-intensity exercise for 12 weeks	HDRS and BDI- depression	Post intervention	Aerobic training and strength training groups showed significant reductions in depressive symptoms
Ojha and Yadav (2016) [31]	yogic techniques	India	Randomised control trial	500 subjects who were retired officials (in the age group of 65–75 years), 250 in each group from municipal areas of 12 towns of eastern Bihar	half an hour daily for six months	Usual activities	Composite psychological wellbeing score – psychological well-being	post intervention	Significant improvement in composite psychological wellbeing score in intervention group
Prakhinkit et al. (2014) [32]	The Buddhist walking meditation program based on aerobic walking exercise incorporating the Buddhist meditations. Traditional walking exercise program involved walking at mild intensity	Thailand	Three arm randomised control trial	Forty-five elderly participants aged 60–90 years with mild-to-moderate depressive symptoms were recruited from university hospital. 15 in each group	Both interventions were performed for 20 min, 3 times/week for 12 weeks	Usual activities	Thai version of GDS (long form)—depression	post intervention	Depression score decreased only in the Buddhist walking meditation group
Shahidi et al. (2011) [33]	laughter yoga (10 sessions) and group exercise program (10 sessions)	Iran	randomised control trial	Seventy depressed old women aged 60–80 years from cultural community of Tehran with GDS score > 10. Laughter Yoga (*n* = 23), exercise therapy (*n* = 23), and control groups (*n* = 24)		No intervention	GDS – depression; Diener life satisfaction scale – Life satisfaction	post intervention	significant decrease in depression scores of both Laughter Yoga and exercise therapy group in comparison to control group. There was no significant difference between Laughter Yoga and exercise therapy groups
Chua and de Guzman (2014) [26]	program consisting of wellness, physical fitness, and livelihood training activities facilitated by volunteer faculty from a local university	Philippines	Randomised control trial	40 community dwelling Filipino elderly aged 60–80 years. Twenty-five subjects were assigned to the intervention group while 15 subjects to the control group	4 months	no intervention	Life Satisfaction Index for the Third Age Short Form (LSITA-SF)—Life satisfaction and GDS—Depression	4 months	The intervention group had significantly higher LSITA-SF scores after the program than before it was implemented and a significant decrease in the depression level
Ghodsbin et al. (2015) [28]	Laughter therapy, including performing breathing and physical exercises as well as laughter techniques	Iran	Randomised control trial	72 senior citizens aged 60 and over referring to Jahandidegan (Khold-e-Barin) retirement community center in Shiraz. With 36 participants in each group	Consists of two 90-min sessions per week over 6 weeks	No intervention	General Health Questionnaire (GHQ-28)	post intervention	significant improvement in mean scores for anxiety but no significant improvement in mean scores for depression in the intervention group compared to the control group
Xu et al. (2016) [35]	Collective exercise intervention that included Baduanjin (Chinese gymnastics) and elderly ballroom dancing	China	randomised control trial	115 elderly hypertensive patients aged 60–70 years old from Fuzhou City, Fujian Province. With 58 participants in Intervention group and 57 in control group	12 weeks	No intervention	Symptom checklist 90- mental disorders and psychological illnesses	post-intervention	After intervention, the Symptom Checklist-90, total score, somatization, obsessive–compulsive symptom, interpersonal sensitivity, depression, anxiety, hostility, and paranoia scores of the intervention group were significantly lower than those of the control group
**Social engagement**									
Aekwarangkoon and Noonil (2020) [35]	weekly positive interpersonal interactions with grandchildren and older adults involving using words of affirmation, spending quality time, offering gifts, performing acts of service and communicating emotional love through physical contacts	Thailand	Cluster randomised control trial	80 older adults aged 60-year and above, living in 4 villages of Thasala District, Nakhon Si Thammarat Province, with 40 older adults in each group	Six weeks	Usual care	Nine-Question Scale and HDRS- depression	at 6^th^, 12th and 24thweek follow-up	a significant decline in Hamilton Rating Scale scores after grandchildren’s love language program
Jacob et al. (2007) [44]	Community based day care which included recreational activities, occupational therapy, counselling services, medical services and a noon meal	India	quasi-experimental, controlled study	41 elderly residents of Pennathur village whose scores were in the lowest third on the socioeconomic status scale and on the social support scale		no intervention	WHO Quality of Life—BREF – Quality of Life	3 months	a significant improvement in quality-of-life scores in those who attended day care compared to those who did not attend (p < 0.001)
Malekafzali et al. (2010) [54]	community mobilization of trained volunteers who were assigned to following tasks: home visits and face to face elderly education, referral to physicians for elderly with health problems, distribution of educational pamphlets, a general meeting question and answer session with the presence of the experts	Iran	pretest–posttest study	200 elderly patient aged 60 years and over			Life satisfaction (No standard validated questionnaires were used)	Post intervention	No significant findings
Rachasrimuang et al. (2018) [39]	Trained youth volunteers were assigned for home visit to the same 6 to 7 elderly persons’ households	Thailand	Cluster randomised control trial	elderly persons, aged 60 years and over living in the study area in 9 villages of Mainapiang Sub-district, Wangyai District, Khon Kaen province	18 weeks	received conventional care by their family and children	Thai version GDS – depression; Thai version of the EQ-5D-5L developed by Mahidol University—Health-related quality of life	9th week and 12th week follow up from baseline measurement	significant reduction in depression scores in intervention group compared to control groups in the 9th-week and 18th-week follow-up. There was significant improvement in self-health perception in overall health status in intervention group compared to control group in the 18th week
**Education**									
Moeini et al. (2020) [9]	Four weekly educational training sessions, each session lasting 60 min comprising of lectures, group discussions, colloquy, booklets and educational pamphlets by experts	Iran	quasi-experimental, controlled study	100 older adults aged 60‐75 years in Hamadan with 40 participants in intervention group and 60 participants in control group		no intervention	Persian version of Oxford Argyle Happiness Inventory – happiness and a questionnaire derived from social support questionnaire	3 months	a significant improvement in the scores of happiness, social support and their components in the intervention group compared to the control group three months after the intervention
Wang et al. (2019) [49]	Mental health lecture and training in a nurse-led Path-oriented Psychological Self-help Intervention	China	quasi-experimental, controlled study	76 empty-nest older adults from 2 districts in the city of Chifeng. 38 in each group	1 month	mental health lecture	Chinese Mental Health Scale (geriatric edition)—mental health status	3 months	The mental health status scores improved in the intervention group 1 month after baseline and sustained for 3 months after the intervention
Yodmai et al. (2021) [10]	Health promotion program that trained family member of older adults to change health behaviours such as eating healthy food, exercising, emotion management and disability preventive activities	Thailand	quasi-experimental, controlled study	Fifty-five older adults aged 60–80 years with chronic diseases, including hypertension, diabetes, hyperlipidaemia, and heart disease in Khon Kaen Province	12 months	usual health promoting activities by health personnel	WHO-Quality of life measurement – Quality of Life; 30-item GDS- depression	postintervention at 9th and 12th months	After the intervention, social support and perception of health care from family members were significantly improved at the 9th month. At the 12th month, overall Quality of life, sensory ability, social participation, intimacy, social support, and perception of health care from family members significantly improved. Depression was also reduced at the 12th month
**Other/Multi-component**									
Abdi et al. (2019) [25]	a religion-spiritual program that included strategies such as reading verses from the Holy Quran and spiritual caring services	Iran	Randomised control trial	100 Older adults with cardiovascular disease from Mostafa–Khomini hospital having a religion of Islam-Shia, 50 older adults in each group	Six educational sessions, each in a week and lasted about 30–45 min	No intervention	BDI– depression; LSI-Z—Life satisfaction	3 months	Higher mean life satisfaction scores and lower mean depression scores in intervention group than control group post intervention
Carandang et al.(2020) [43]	Peer counselling group: Peer counsellors performed 1-h home visits weekly to their assigned clients Social engagement group: Senior citizens joined 3-h weekly social events held at the OSCA Center Combination group: both peer counselling and social engagement interventions	Philippines	4-arm quasi-experimental, controlled study	270 community-dwelling Filipino senior citizens with mean age was 68.3 years, who had tendency towards depression based on the 15-item Geriatric Depression Scale. peer counselling (*n* = 65), social engagement (*n* = 66), and combination (*n* = 65) and control group (*n* = 68)	3 months	usual or standard care from health and aged care services	GDS—Depression. 8-item UCLA Loneliness Scale—Loneliness	3 months	Social engagement and combined intervention had a large effect on reducing depressive symptoms while peer counselling had only moderate effect. All interventions had only small effect on improving loneliness
Ebrahimi et al. (2020) [27]	In one group, older adults received intergenerational programs plus aerobic exercises in the presence of young adults, and in other group they received intergenerational programs only	Iran	Three arm randomised control trial	150 older adults (mean age, 71.4 years) and 100 students (mean age, 21.8 years) living in Mashhad	8 weeks	Daily routine activities	WHO Quality of Life, BREF – Quality of Life	postintervention at 8 weeks from baseline assessment	a significant difference in the mean scores of quality-of-life dimensions between the three groups
Zhan et al. (2018) [50]	mental health services including knowledge about healthy mental state, psychological consultation/treatment, and access to a psychiatric hotline	China	quasi-experimental, controlled study	2,000 elderly residents, aged 60 years and above in the Longhua sub-district of Shanghai	1 year	Only the basic mental health services	Generalized Anxiety Disorder 7-item scale – Anxiety and depression; PHQ-9 and Quality of Life Index – quality of life and General Well-Being Schedule (GWB) – wellbeing	6 months and 12 months	PHQ-9 and GAD-7 scores gradually decreased and GWB score gradually increased in intervention group. After 12 months, compared the control group, the scores of subscales in GWB satisfaction and interest in life, worries about health, depressed versus cheerful mood, and relaxation versus tension (anxiety) were significantly better
Rana et al. (2009) [45]	Community based intervention that included physical activity, advice on healthy food intake and other aspects of management. Social awareness was provided by means of information about the contribution of and challenges faced by older adults at home and the community, including information about older adults’ health and health care. Intervention activities provided to older adults, caregivers, household members and community people	Bangladesh	quasi-experimental, controlled study	839 elderly persons (≥ 60 years) eight randomly selected villages (Intervention: *n* = 4; Control: *n* = 4) in rural Bangladesh. 425 elderly persons in the intervention group and 414 in the control group	15 months	No intervention	Health related quality of life – generic instrument	3 months after intervention	significant differences noted in the physical, social, spiritual, environment and overall Health related quality of life
Zhang et al. (2021) [37]	Self-Mutual-Group based intervention, which consisted of three stages: self-management (2 months), mutual management (2 months), and group-management (3 months)	China	randomised control trial	396 empty-nest older adults in Taiyuan, Shanxi. With 204 participants in the intervention group and 192 in control group	7 months	No intervention	Short Form 36-Item Health Survey – Quality of Life	postintervention (7th month)	After the intervention, participants’ scores on Mental Component Summary, Physical Component Summary, role emotional, vitality, social function, mental health and general health increased significantly in the intervention group
Li et al. (2020) [56, 64]	The Collaborative Care for Older People with Comorbid Hypertension and Depression (COACH) model integrates the care provided by the older person's primary care provider (PCP) with that delivered by an Aging Worker (AW) from the village's Aging Association, supervised by a psychiatrist consultant	China	Five focus groups: two with VDs, two with AWs, and one with psychiatrists				iterative process		Facilitators to implementation include training, leaders’ support, geographic proximity between VD and AW pairs, pre-existing relationships among care team members, comparability of COACH activities and existing practices of VDs and AWs, and care team members’ caring about older members of their villages. Barriers to sustainability include frustration of some VDs related to their low wages and feelings of overload of some AWs

*Legend.* *DASS* Depression Anxiety Stress Scales, *GDS* Geriatric depression scale, *WHO* World Health Organization, *LSI* Life Satisfaction Index A, *HDRS* Hamilton Depression Rating Scale, *BDI* Beck Depression Inventory, *OHQ* Oxford Happiness Questionnaire.

The interventions were predominantly delivered by health or social care professionals (*n* = 11) [8, 9, 26, 30, 34, 40, 46–50, 53], trained lay volunteers (*n* = 5) [39, 54, 56, 57, 60] and research team members with a lack of qualification description [10, 21, 22, 25, 29, 31, 33, 35–38, 41, 45]. In the Carandang et al. [43] study, the intervention involved the older adults themselves participating in a volunteer activity (assist in behaviour change and engaging with community) whereas Borbon-Castro et al. [42] involved Higher Education students in physical activity and sport discipline. In the remaining studies, there were no information about who delivered the intervention [7, 27, 28, 32, 44, 51, 52, 58].

Twenty-six studies [7, 8, 10, 21, 22, 25, 26, 28, 29, 32–35, 38–40, 42, 43, 46–49, 51, 53, 55, 57] reported depression as an outcome variable. Other outcomes included anxiety [53], post-traumatic stress disorder [8], happiness [9, 34], life satisfaction [26], quality of life [41], stress [51], mental health status [49], loneliness [36], resilience [43] and social support [9].

### Quality of assessed studies

**Fig. 2:**
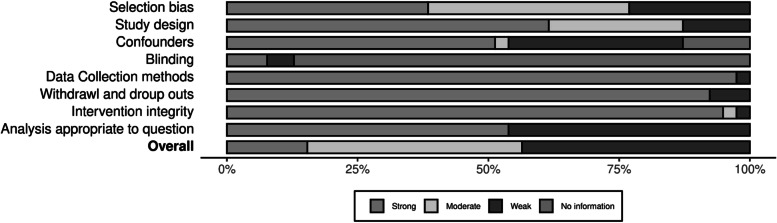
Component ratings about each component of quality assessment tool presented as percentages across all included studies

**Fig. 3:**
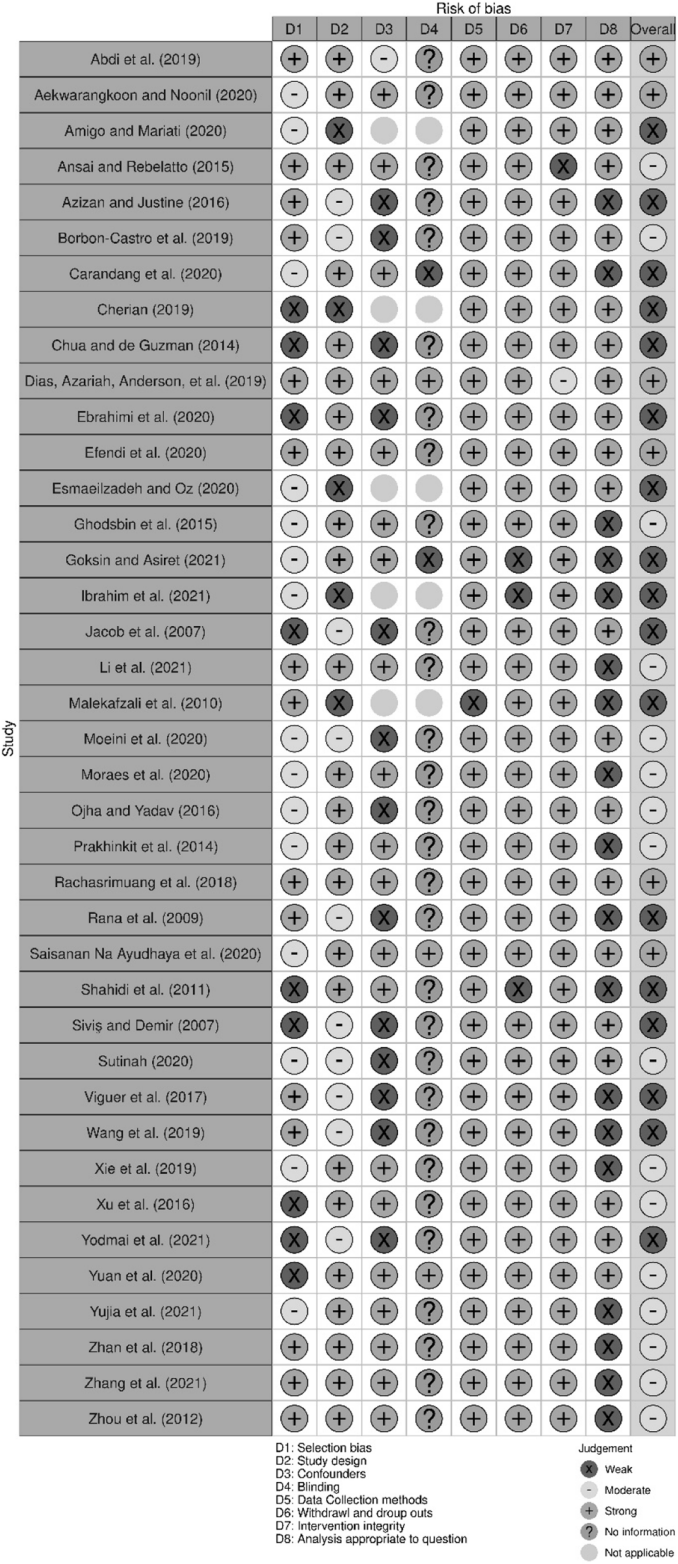
Study quality rating of each component of each study

Figures 2 and 3 provide an overview of the summary ratings for each risk of bias item. Of the quantitative studies, overall, six studies were rated strong [8, 25, 38, 39, 46, 55], 16 studies [9, 21, 22, 28, 30–32, 34–37, 40, 42, 47, 50, 57] were rated moderate, and 17 studies [7, 10, 26, 27, 29, 33, 41, 43–45, 48, 49, 51–54, 58] rated weak. Twenty studies reported similarity in comparison groups at baseline and were rated strong for confounders domain. Data collection methods, withdrawal and dropouts and intervention integrity were reported appropriately in most of the studies and were therefore rated strong for these domains. Regarding the four qualitative studies (see Table 2), ethics were missing for some, and limited learning was suggested.

**Table 2:** Risk of bias ratings for qualitative studies using the Critical Appraisal Skills Programme (CASP) for Qualitative Studies

Criteria	Azariah et al. (2019)	Li et al. (2020)	Sivis & Demir (2007)	Tang et al. (2015)
1. Was there a clear statement of the aims of the research?	Yes	Yes	Yes	Yes
2. Is a qualitative methodology appropriate?	Yes	Yes	Yes	Yes
3. Was the research design appropriate to address the aims of the research?	Yes	Yes	Yes	Yes
4. Was the recruitment strategy appropriate to the aims of the research?	Yes	Yes	Yes	Yes
5. Was the data collected in a way that addressed the research issue?	Yes	Yes	Yes	Yes
6. Has the relationship between researcher and participants been adequately considered?	No	No	No	No
7. Have ethical issues been taken into account?	Yes	No	No	No
8. Was the data analysis sufficiently rigorous?	Yes	Yes	No	Yes
9. Is there a clear statement of findings?	Yes	No	No	Yes
10. How valuable is the research?	Valuable	Limited	Limited	Valuable

### Groupings of interventions

**Fig. 4:**
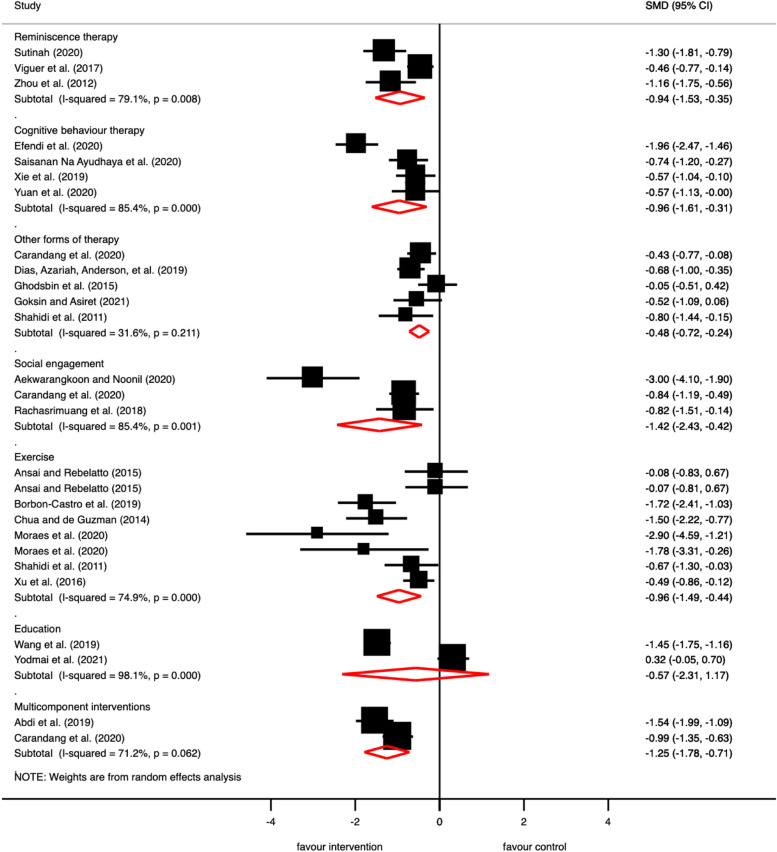
Forrest Plot of studies including data on outcomes of levels of depressive symptomatology. Legend. Two comparisons from RCTs [Ansai and Reblatto (2015) and Moares et al. (2020)] with three treatment arms have been included under the category exercise interventions. The number of participants in the control group in these comparisons has been halved so as to avoid double counting. Black boxes represent the effect estimates (standardised mean difference), and the horizontal bars are for the 95% confidence intervals (CIs). The diamond is for the pooled effect estimate and 95% CI and the dotted vertical line centered on the diamond has been added to assist visual interpretation

We categorised the 40 included studies into the following categories (and sub-categories) (see Table 1): (1) Therapy interventions (Sub-categories: Established forms of short-term therapy, Other forms of therapy); (2) Exercise interventions (Sub-categories: Muscle strengthening, Relaxation exercise); (3) Educational interventions; (4) Social engagement interventions; (5) Multi-component. Some interventions could be fitted under two categories, with some interventions also being multi-component. Figure 4 shows the Forrest plot of studies including data on outcomes of levels of depressive symptomatology.

#### Psychological therapy interventions

##### Established forms of therapy

Eleven studies evaluated the effectiveness of established forms of therapy interventions, including cognitive behavioural therapy [8, 34, 46, 57] and reminiscence therapy [7, 30, 36, 40, 47, 48, 58]. Cognitive behaviour therapy (CBT) was delivered by trained therapist [8, 34], trained health care workers [57] and a team comprising of trained healthcare workers and trained volunteers [46]. In one study [8], the focus of CBT was trauma due to recent earthquake, in another study the CBT was tailored to local context such as encouraging to visit the temple regularly [57]. Two studies focused on the behavioural activation component of CBT [34, 46], where participants were encouraged to engage in activities that they enjoyed. The participants themselves selected and planned their activities for the duration of intervention.

Similarly, reminiscence therapy aimed to restore beliefs and acquire coping skills among older adults by recalling past events and memories. All studies implemented reminiscence therapy in groups and one study based reminiscence therapy on traditional festivals [36]. Some studies combined reminiscence therapy with other interventions like exercise [36] and psychoeducation [47]. Two studies were rated strong, six studies moderate, and three studies weak.

Xie et al. [34] reported reductions in anxiety scores following behaviour activation. However, another study [46] reported reduction in anxiety scores at 3-month follow-up statistically significant improvement was not sustained at 6-months follow-up. Similarly, CBT did not have a significant effect on anxiety scores [57]. The studies reported reduction in PTSD scores [8], improvement in happiness scores [34], reduction in stress and loneliness [30, 36], improvement in resilience score [36] and mixed results for life satisfaction [48, 58] and quality of life scores [8, 57] with one study showing improvement and others showing no significant effect.

The pooled estimate of three studies [40, 47, 48] showed a moderate to large reduction in depression scores in older adults following reminiscence therapy (SMD -0.94, 95% CI -1.53 to -0.35, *I*^*2*^ = 79.1%) with considerable heterogeneity. Of the remaining studies, one study [7] reported depression as an outcome. This study also reported a significant reduction in depression scores in older adults following reminiscence therapy. The pooled analysis of four studies assessing cognitive behaviour therapy showed a nonsignificant reduction in depression scores following intervention (SMD -0.96, 95% CI -1.61 to 0.31, *I*^*2*^ = 85.4%) with considerable heterogeneity.

Qualitative findings showed that older adults experienced reminiscence therapy to add a new perspective to their past experiences, strengthened their social connectedness and provided psychological support [58]. In another study [57, 59], facilitators to delivering a CBT intervention by village doctors included previous knowledge of mental health issues, policy alliance, instruction manuals and traning in CBT techniques. Busy schedules and lack of comptency in delivering the intervention were reported to be barriers.

##### Other forms of therapy

Seven studies evaluated the effectiveness of other forms of therapy interventions, including music therapy [51], progressive muscle relaxation [29], problem solving therapy [55], psychosocial care [52], laughter therapy [28, 33], and peer counselling [43].

Problem solving therapy was delivered by trained lay counsellors in depression in later life [55, 61]. Participants in the study received instructions for management of their chronic conditions, encouraged to access health care services and assist in obtaining available resources [55]. Similarly, peer counselling was delivered by volunteers trained to interact with older adults, identify their problems and encourage them to adopt healthy lifestyle [43]. Laughter therapy was delivered as an integrated intervention of laughter exercise, breathing exercise and physical exercise [28, 33]. One study was rated strong, one moderate and five studies weak.

Studies reported improvement in life satisfaction [33], improvement in quality of life [52], resilience [43], social support [43] and mixed results for loneliness with one study showing improvement in loneliness score [52] and other study showing no effect [43]. The pooled estimate of five studies [28, 29, 33, 43, 55] showed a small to moderate reduction in depression scores in older adults following other forms of therapy (SMD -0.48, 95% CI -0.72 to -0.24, *I*^*2*^ = 31.6%) with likely no important heterogeneity. Only one study [52] reported depression as an outcome, but demonstrated no significant reduction in depression at post intervention follow up.

Qualitative findings from one study [55, 61] reported that older adults enjoyed the intervention as it provided them with the opportunity to share their feelings, which helped coping with loneliness and isolation.

#### Exercise interventions

Eleven studies evaluated the effectiveness of exercise interventions. These included aerobic exercises [21, 22, 32, 33, 42], muscle strengthening exercise [21, 22, 26, 35, 41, 42, 53] and yoga [31]. Aerobic exercises included exercise on stationary bikes [21, 22] or treadmills [22], walking [32, 42], jogging [53] and stretching [53]. Similarly, muscle strengthening exercise included weightlifting [21, 22], resistance training [21, 42], self-loading [42], physical fitness training [26] Chinese gymnastics and ballroom dancing [35]. Only one study [53] used a virtual medium and video recording to deliver the exercise intervention to older adults. Seven studies were rated as moderate, and four studies as weak. Studies reported improvement in quality of life [41], no effect on anxiety [53] and mixed results for life satisfaction scores with one study reporting improvement [26] but the other study reported no effect [33].

Six studies [21, 22, 26, 33, 35, 42] reported data that could be pooled in a meta-analysis. The pooled estimate of six studies showed a moderate to large reduction in depression scores in older adults following exercise intervention (SMD -0.96, 95% CI -1.49 to -0.44, *I*^*2*^ = 74.9%) with substantial heterogeneity. Out of the remaining studies three studies that reported depression as an outcome [32, 41, 53], two studies [32, 41] reported significant reduction in depression scores following the exercise intervention. However, one study [53] reported non-significant effect of exercise intervention on depression scores.

#### Educational interventions

Three studies evaluated the effectiveness of educational interventions, including educational training sessions [9, 49] and health promotion programs [10]. Studies used various techniques to provide skills and knowledge to older adults such as lectures/presentation [9, 49], printed material [9, 49], training [10, 49], self-study [49], and discussion [9, 49]. These interventions covered a range of topics including basic knowledge about mental health, exercise, healthy diet, sleep hygiene and emotion regulation [9, 10, 49]. One study was rated moderate, and two studies were rated weak. Reported outcomes covered improvement in happiness [9], social support [9], mental health status [49] and quality of life [10] scores. Pooled analysis of two studies [10, 49] showed a non-significant reduction of depression scores following education interventions (SMD -0.57, 95% CI -2.31 to 1.17, *I*^*2*^ = 98.1%) with considerable heterogeneity.

#### Social engagement interventions

Six studies evaluated the effectiveness of social engagement interventions, including intergenerational programmes [27, 38, 39], community-based day care [44], home visits by trained local volunteers [54] and social engagement groups [43]. Intergenerational programmes engaged older adults with younger generations in activities to facilitate physical activity and social connectedness among older adults [27, 38, 39]. Similar objectives were also incorporated in a community-based day care intervention that offered activities such as leisure exercise, counselling, medical services, and lunch [44]. Two studies were rated strong, and four studies were rated weak.

The studies reported improvement in quality of life [27, 39, 44], loneliness [43], resilience [43], social support [43] but no effect on life satisfaction scores [54]. The pooled estimate of three studies [38, 39, 43] showed a moderate to large reduction in depression scores in older adults following social engagement interventions (SMD -1.42, 95% CI -2.43 to -0.42, *I*^*2*^ = 85.4%) with substantial heterogeneity.

#### Multicomponent interventions

Seven studies evaluated the effectiveness of multicomponent interventions, involving for example peer counselling plus social engagement groups [43], intergenerational programs plus aerobic exercise [27], exercise combined with dietary counselling and social engagement [45]. One study was rated strong, two studies moderate, three studies weak, and one study was assessed only for a qualitative section. The studies reported improvement in life satisfaction [25], quality of life [27, 37, 45] anxiety [50] resilience [43], social support [43] but no improvement in loneliness [43] scores.

The pooled estimate of two studies [25, 43] showed a moderate to large reduction in depression scores in older adults following multicomponent intervention (SMD -1.25, 95% CI -1.78 to -0.71, *I*^*2*^ = 71.2%) with substantial heterogeneity. Findings from the qualitative section of a single study [56] reported health care providers approved the multidisciplinary team-based approach comprising village doctors, ageing workers, and psychiatrists for providing integrated health care including promotion of mental health. The intervention was also favoured by health care providers as it could be easily integrated into their existing practices.

## Discussion

This is the first systematic review and meta-analysis to explore the different types, components, and effects of community-based psychosocial interventions for older adults living in LMICs. With older adults one of the fastest growing population groups globally [6], adequate support needs to be in place not only for their physical health, but also for their mental health and well-being. Across 12 different countries, five types of interventions with varied degrees of effectiveness emerged, including established forms of therapies such as reminiscence therapy, exercise interventions, educational interventions, those focusing on social engagement, as well as multi-component interventions. There was substantial heterogeneity in all the pooled effect estimates except for other forms of therapy. However, we could not investigate the heterogeneity with subgroup or meta regression due to small number of studies in each meta-analysis. The other forms of therapy resulted in small to moderate reduction in depression scores (SMD -0.48, 95% CI -0.72 to -0.24) in older adults. In an era of an ongoing pandemic, affecting people’s mental health across the globe [65], these different types of interventions may provide some ways to alleviate older adults’ poor mental health, if adequately tailored to their needs.

The included studies illustrate not only different types and components of interventions, but also different approaches to their delivery. Interventions were provided one-on-one or in group settings, with older adults meeting in different settings to receive the intervention, including the Office for Senior Citizens Affairs [43], at their homes [38], or welfare centres [28]. The way in which an intervention is delivered can affect the level of adherence or enrolment with some preferring group settings and the additional social aspect and others individual engagement. The provider of the intervention also varied, ranging from different types of professionals to volunteers. These variations in intervention mechanisms (type, components, delivery) make it difficult to directly compare interventions. Moreover, the varied contextual factors (cultural, geographical, and population) create further barriers for direct comparison. However, as shown, there are some types of interventions which can be categorised despite these differences, providing an overview of existing psychosocial interventions for older adults in LMICs, and highlighting different approaches in conducting these interventions.

Overall, the majority of interventions were found to be initially effective, with 14 studies being RCTs, although these effects were not always maintained at follow-up, however more rapid recovery is important to people with problems and their close associates. Where improvements in mental health or quality of life or reductions in loneliness were found, some differences were statistically significant, but not all. With depression being the most commonly reported outcome measure, exercise and social engagement indicated greater reductions in depressive symptoms in treatment groups [22, 43] as opposed to established forms of therapies or education for example [10, 46]. However, this conclusion is tempered given the varied tools used to measure depressive symptoms. These outcomes were not able to be assessed via the same quantitative approach in our meta-analysis however due to limitations among reported outcomes and inconsistent use of established outcome measures. Considering the population participating in these interventions are older adults residing in a variety of LMIC, it is unsurprising to find varied outcome measures, with some tools being culturally adapted and validated in some countries but not others. This reflects the imperative that such interventions need to be adapted to the cultural and population contexts, and may require different outcome measures or forms of evaluations than standardised tools to fully capture the effects of receiving an educational or exercise intervention for example. Whilst this increases local study validity and reliability, it does limit the potential for detailed quantitative cross-country comparisons.

Whilst some interventions were delivered one-on-one and others in group settings, improved well-being and mental health are linked to socialising with others [66] so that even one-on-one interventions consider widening and strengthening a person’s social circle. Interventions specifically focusing on social engagement reported reductions in loneliness and improvements in social support, quality of life, and resilience. One of the two most effective interventions in reducing depression, based on the meta-analysis, was a study on social engagement by Aekwarangkoon et al. [38]. Considering the relationship between loneliness and social isolation and poor mental health [67], which is of particular concern in many older adults who are living alone given its frequency and links to cognitive deteriorations [68], social engagement-focused interventions appear to be an important way of supporting older adults’ mental health and well-being. An additional benefit of these types of interventions is that they outlast the intervention delivery time, by enabling social networks which can be drawn upon and integrated into a person’s everyday life subsequently. This is particularly pertinent to these geographical and cultural contexts as mental health support in general is sparse and difficult to access, if not prohibited by stigma [69], so that easily achievable changes to people’s daily lives which do not specifically label attendees with mental health may be more suitable. In contrast, time-limited interventions such as CBT and reminiscence therapy may have a time-restricted impact, so that future research should follow up participants of social interventions for longer and establish the potential long-term integration and influence of the initial intervention into their daily lives.

### Limitations

Concerning the limitations of included studies, many reported only limited methodological details about the interventions and population contexts, making it more difficult to compare findings. This is reflected in the low-quality ratings. Moreover, studies used different outcomes and outcome measures, further enhancing the complexity of comparing intervention results.

Concerning the review and meta-analysis, there are some limitations to consider. Whilst studies emerged from 12 LMIC and from across different geographical regions, and overall included a large number of reported interventions, findings indicate how different older adult populations have been supported by interventions for mental health and well-being in different contexts without being widely representative of all LMIC. Even with countries that reported an intervention, there are variations within countries, so that an intervention reported from the North of India for example may not be as relevant, acceptable and/or effective if it had been conducted in the South. This is not surprising as cultural, socio-economic, and other differences may be considerable across large populations in different areas. It is a deficit of such research to make assumptions that coalesce very different communities within a summarised country commentary [70], and suggests that interventions need to be adapted to localised contexts. Based on these variations in types of interventions and contexts, we were only able to conduct a meta-analysis on depression and no other outcome measures such as anxiety or quality of life. For multi-centre interventions to be comparable, the same outcome measures, linguistically and culturally adapted to the localised population, need to be employed to make more valid comparisons between intervention effects.

## Conclusions

Psychosocial interventions for older adults living in LMIC are highly varied and have different levels of efficacy. Interventions may provide specific elements which can be adapted, depending on the different needs and local contexts of the relevant populations. These different intervention types and their components can form the foundation and provide a menu for the development or adaptation of different psychosocial community-based interventions supporting underserved older adult populations in LMIC. In the ongoing pandemic and increased mental health problems in the general population and older adults, including in LMIC [65], developing and implementing sustainable and effective psychosocial interventions to support good mental health are ever more important. To ensure improved comparability across interventions, there is a need for multi-country and -setting comparisons using the same outcome measures, as well as longer follow-up times beyond the lifespans of interventions.

## Supplementary Information

**Additional file 1: Appendix 1.** Search strategy.

## Data Availability

The datasets used and/or analysed during the current study are available from the corresponding author on reasonable request.

## Abbreviation

LMIC: Lower- and middle-income countries
