# Supplementary material for: Community-based mental health and well-being interventions for older adults in low- and middle-income countries: a systematic review and meta-analysis
Source: BMC Geriatr. 2022 Sep 29;22:773. doi: 10.1186/s12877-022-03453-1 (PMC9520120; doi:10.1186/s12877-022-03453-1)
Supplement: Supplementary file 1 — Additional file 1: Appendix 1. Search strategy. [file 12877_2022_3453_MOESM1_ESM.docx]

**Appendix I. Search strategy**

“mental illness” [tiab] OR “mental health” [MeSH] OR “mental disorders” [MeSH] OR “major depressive disorder” [tiab] OR “depression” [tiab] OR “anxiety” [tiab] OR “stress disorder” [tiab] OR wellbeing [tiab] OR resilience [tiab] OR “suicidal behaviour” [tiab] OR “psychological distress” [tiab]

AND

((“clinical trial” [MeSH] OR psychosicial intervention [MeSH] OR preventi* [tiab] OR interven*[tiab] OR implement*[tiab] OR program*[tiab] OR impact[tiab] OR impacts[tiab] OR effect*[tiab]) OR (barrier*[tiab] OR hurdle[tiab] OR obstruct*[tiab] OR promot*[tiab] OR facilitator*[tiab] OR support*[tiab] OR encourag*[tiab] “Coping skills” [tiab]))

AND

Elderly[tiab] OR Senior[tiab] OR seniors[tiab] OR older adult[tiab] OR older adults[tiab] OR elderly[tiab] OR geriatric[tiab] OR geriatrics[tiab] OR old people[tiab] OR older‐age[tiab] OR "aged"[Mesh] OR "aged, 80 and over"[Mesh] OR old age[tiab] OR older people[tiab]

AND

afghanistan[tiab] OR albania[tiab] OR algeria[tiab] OR american samoa[tiab] OR angola[tiab] OR antigua[tiab] OR barbuda[tiab] OR argentina[tiab] OR armenia[tiab] OR armenian[tiab] OR aruba[tiab] OR azerbaijan[tiab] OR bahrain[tiab] OR bangladesh[tiab] OR barbados[tiab] OR belarus[tiab] OR byelarus[tiab] OR belorussia[tiab] OR byelorussian[tiab] OR belize[tiab] OR british honduras[tiab] OR benin[tiab] OR dahomey[tiab] OR bhutan[tiab] OR bolivia[tiab] OR bosnia[tiab] OR herzegovina[tiab] OR botswana[tiab] OR bechuanaland[tiab] OR brazil[tiab] OR brasil[tiab] OR bulgaria[tiab] OR burkina faso[tiab] OR burkina fasso[tiab] OR upper volta[tiab] OR burundi[tiab] OR urundi[tiab] OR cabo verde[tiab] OR cape verde[tiab] OR cambodia[tiab] OR kampuchea[tiab] OR khmer republic[tiab] OR cameroon[tiab] OR cameron[tiab] OR cameroun[tiab] OR central african republic[tiab] OR ubangi shari[tiab] OR chad[tiab] OR chile[tiab] OR china[tiab] OR colombia[tiab] OR comoros[tiab] OR comoro islands[tiab] OR mayotte[tiab] OR congo[tiab] OR zaire[tiab] OR costa rica[tiab] OR cote d’ivoire[tiab] OR cote d’ ivoire[tiab] OR cote divoire[tiab] OR cote d ivoire[tiab] OR ivory coast[tiab] OR croatia[tiab] OR cuba[tiab] OR cyprus[tiab] OR czech republic[tiab] OR czechoslovakia[tiab] OR djibouti[tiab] OR french somaliland[tiab] OR dominica[tiab] OR dominican republic[tiab] OR ecuador[tiab] OR egypt[tiab] OR united arab republic[tiab] OR el salvador[tiab] OR equatorial guinea[tiab] OR spanish guinea[tiab] OR eritrea[tiab] OR estonia[tiab] OR eswatini[tiab] OR swaziland[tiab] OR ethiopia[tiab] OR fiji[tiab] OR gabon[tiab] OR gabonese republic[tiab] OR gambia[tiab] OR georgia[tiab] OR georgian[tiab] OR ghana[tiab] OR gold coast[tiab] OR gibraltar[tiab] OR greece[tiab] OR grenada[tiab] OR guam[tiab] OR guatemala[tiab] OR guinea[tiab] OR guyana[tiab] OR guiana[tiab] OR haiti[tiab] OR hispaniola[tiab] OR honduras[tiab] OR hungary[tiab] OR india[tiab] OR indonesia[tiab] OR timor[tiab] OR iran[tiab] OR iraq[tiab] OR isle of man[tiab] OR jamaica[tiab] OR jordan[tiab] OR kazakhstan[tiab] OR kazakh[tiab] OR kenya[tiab] OR korea[tiab] OR kosovo[tiab] OR kyrgyzstan[tiab] OR kirghizia[tiab] OR kirgizstan[tiab] OR kyrgyz republic[tiab] OR kirghiz[tiab] OR laos[tiab] OR lao pdr[tiab] OR lao people's democratic republic[tiab] OR latvia[tiab] OR lebanon[tiab] OR lesotho[tiab] OR basutoland[tiab] OR liberia[tiab] OR libya[tiab] OR libyan arab jamahiriya[tiab] OR lithuania[tiab] OR macau[tiab] OR macao[tiab] OR macedonia[tiab] OR madagascar[tiab] OR malagasy republic[tiab] OR malawi[tiab] OR nyasaland[tiab] OR malaysia[tiab] OR maldives[tiab] OR indian ocean[tiab] OR mali[tiab] OR malta[tiab] OR micronesia[tiab] OR kiribati[tiab] OR marshall islands[tiab] OR nauru[tiab] OR northern mariana islands[tiab] OR palau[tiab] OR tuvalu[tiab] OR mauritania[tiab] OR mauritius[tiab] OR mexico[tiab] OR moldova[tiab] OR moldovian[tiab] OR mongolia[tiab] OR montenegro[tiab] OR morocco[tiab] OR ifni[tiab] OR mozambique[tiab] OR portuguese east africa[tiab] OR myanmar[tiab] OR burma[tiab] OR namibia[tiab] OR nepal[tiab] OR netherlands antilles[tiab] OR nicaragua[tiab] OR niger[tiab] OR nigeria[tiab] OR oman[tiab] OR muscat[tiab] OR pakistan[tiab] OR panama[tiab] OR papua new guinea[tiab] OR paraguay[tiab] OR peru[tiab] OR philippines[tiab] OR philipines[tiab] OR phillipines[tiab] OR phillippines[tiab] OR poland[tiab] OR polish people's republic[tiab] OR portugal[tiab] OR portuguese republic[tiab] OR puerto rico[tiab] OR romania[tiab] OR russia[tiab] OR russian federation[tiab] OR ussr[tiab] OR soviet union[tiab] OR union of soviet socialist republics[tiab] OR rwanda[tiab] OR ruanda[tiab] OR samoa[tiab] OR pacific islands[tiab] OR polynesia[tiab] OR samoan islands[tiab] OR sao tome and principe[tiab] OR saudi arabia[tiab] OR senegal[tiab] OR serbia[tiab] OR seychelles[tiab] OR sierra leone[tiab] OR slovakia[tiab] OR slovak republic[tiab] OR slovenia[tiab] OR melanesia[tiab] OR solomon island[tiab] OR solomon islands[tiab] OR norfolk island[tiab] OR somalia[tiab] OR south africa[tiab] OR south sudan[tiab] OR sri lanka[tiab] OR ceylon[tiab] OR saint kitts and nevis[tiab] OR st kitts and nevis[tiab] OR saint lucia[tiab] OR st lucia[tiab] OR saint vincent[tiab] OR st vincent[tiab] OR grenadines[tiab] OR sudan[tiab] OR suriname[tiab] OR surinam[tiab] OR syria[tiab] OR syrian arab republic[tiab] OR tajikistan[tiab] OR tadjikistan[tiab] OR tadzhikistan[tiab] OR tadzhik[tiab] OR tanzania[tiab] OR tanganyika[tiab] OR thailand[tiab] OR siam[tiab] OR timor leste[tiab] OR east timor[tiab] OR togo[tiab] OR togolese republic[tiab] OR tonga[tiab] OR trinidad[tiab] OR tobago[tiab] OR tunisia[tiab] OR turkey[tiab] OR turkmenistan[tiab] OR turkmen[tiab] OR uganda[tiab] OR ukraine[tiab] OR uruguay[tiab] OR uzbekistan[tiab] OR uzbek[tiab] OR vanuatu[tiab] OR new hebrides[tiab] OR venezuela[tiab] OR vietnam[tiab] OR viet nam[tiab] OR middle east[tiab] OR west bank[tiab] OR gaza[tiab] OR palestine[tiab] OR yemen[tiab] OR yugoslavia[tiab] OR zambia[tiab] OR zimbabwe[tiab] OR northern rhodesia[tiab] OR global south[tiab] OR africa south of the sahara[tiab] OR sub saharan africa[tiab] OR subsaharan africa[tiab] OR central africa[tiab] OR north africa[tiab] OR northern africa[tiab] OR magreb[tiab] OR maghrib[tiab] OR sahara[tiab] OR southern africa[tiab] OR east africa[tiab] OR eastern africa[tiab] OR west africa[tiab] OR western africa[tiab] OR west indies[tiab] OR indian ocean islands[tiab] OR caribbean[tiab] OR central america[tiab] OR latin america[tiab] OR south america[tiab] OR central asia[tiab] OR north asia[tiab] OR northern asia[tiab] OR southeastern asia[tiab] OR south eastern asia[tiab] OR southeast asia[tiab] OR south east asia[tiab] OR western asia[tiab] OR east europe[tiab] OR eastern europe[tiab] OR developing country[tiab] OR developing countries[tiab] OR developing nation[tiab] OR developing nations[tiab] OR developing population[tiab] OR developing populations[tiab] OR developing world[tiab] OR less developed country[tiab] OR less developed countries[tiab] OR less developed nation[tiab] OR less developed nations[tiab] OR less developed world[tiab] OR lesser developed countries[tiab] OR lesser developed nations[tiab] OR under developed country[tiab] OR under developed countries[tiab] OR under developed nations[tiab] OR under developed world[tiab] OR underdeveloped country[tiab] OR underdeveloped countries[tiab] OR underdeveloped nation[tiab] OR underdeveloped nations[tiab] OR underdeveloped population[tiab] OR underdeveloped populations[tiab] OR underdeveloped world[tiab] OR middle income country[tiab] OR middle income countries[tiab] OR middle income nation[tiab] OR middle income nations[tiab] OR middle income population[tiab] OR middle income populations[tiab] OR low income country[tiab] OR low income countries[tiab] OR low income nation[tiab] OR low income nations[tiab] OR low income population[tiab] OR low income populations[tiab] OR lower income country[tiab] OR lower income countries[tiab] OR lower income nations[tiab] OR lower income population[tiab] OR lower income populations[tiab] OR underserved countries[tiab] OR underserved nations[tiab] OR underserved population[tiab] OR underserved populations[tiab] OR under served population[tiab] OR under served populations[tiab] OR deprived countries[tiab] OR deprived population[tiab] OR deprived populations[tiab] OR poor country[tiab] OR poor countries[tiab] OR poor nation[tiab] OR poor nations[tiab] OR poor population[tiab] OR poor populations[tiab] OR poor world[tiab] OR poorer countries[tiab] OR poorer nations[tiab] OR poorer population[tiab] OR poorer populations[tiab] OR developing economy[tiab] OR developing economies[tiab] OR less developed economy[tiab] OR less developed economies[tiab] OR underdeveloped economies[tiab] OR middle income economy[tiab] OR middle income economies[tiab] OR low income economy[tiab] OR low income economies[tiab] OR lower income economies[tiab] OR low gdp[Tiab] OR low gnp[tiab] OR low gross domestic[tiab] OR low gross national[tiab] OR lower gdp[tiab] OR lower gross domestic[tiab] OR lmic[tiab] OR lmics[tiab] OR third world[tiab] OR lami country[tiab] OR lami countries[tiab] OR transitional country[tiab] OR transitional countries[tiab] OR emerging economies[tiab] OR emerging nation[tiab] OR emerging nations[tiab]

NOT

(animals [mh] NOT humans [mh])
